# Modulation of miR-145-5p and miR-146b-5p levels is linked to reduced parasite load in H9C2 *Trypanosoma cruzi* infected cardiomyoblasts

**DOI:** 10.1038/s41598-022-05493-4

**Published:** 2022-01-26

**Authors:** Priscila Silva Grijó Farani, Beatriz Iandra Silva Ferreira, Daniel Gibaldi, Joseli Lannes-Vieira, Otacilio Cruz Moreira

**Affiliations:** 1 Real Time PCR Platform RPT09A, Laboratory of Molecular Biology and Endemic Diseases, Oswaldo Cruz Institute, Oswaldo Cruz Foundation, Rio de Janeiro, Brazil; 2 Laboratory of Biology of the Interactions, Oswaldo Cruz Institute, Oswaldo Cruz Foundation, Rio de Janeiro, Brazil

**Keywords:** Parasite host response, Mechanisms of disease

## Abstract

In the heart tissue of acutely *Trypanosoma cruzi*-infected mice miR-145-5p and miR-146b-5p are, respectively, downregulated and upregulated. Here, we used the H9C2 rat cardiomyoblast cell line infected with the Colombian *T. cruzi* strain to investigate the parasite-host cell interplay, focusing on the regulation of miR-145-5p and miR-146b-5p expression. Next, we explored the effects of interventions with the trypanosomicidal drug Benznidazole (Bz) alone or combined with Pentoxifylline (PTX), a methylxanthine derivative shown to modulate immunological and cardiac abnormalities in a model of chronic chagasic cardiomyopathy, on parasite load and expression of miR-145-5p and miR-146b-5p. The infection of H9C2 cells with trypomastigote forms allowed parasite cycle with intracellular forms multiplication and trypomastigote release. After 48 and 144 h of infection, upregulation of miR-145-5p (24 h: 2.38 ± 0.26; 48 h: 3.15 ± 0.9-fold change) and miR-146b-5b (24 h: 2.60 ± 0.46; 48 h: 2.97 ± 0.23-fold change) was detected. The peak of both miRNA levels paralleled with release of trypomastigote forms. Addition of 3 µM and 10 µM of Bz 48 h after infection reduced parasite load but did not interfere with miR-145-5p and miR-146b-5p levels. Addition of PTX did not interfere with Bz-induced parasite control efficacy. Conversely, combined Bz + PTX treatment decreased the levels of both microRNAs, resembling the expression levels detected in non-infected H9C2 cells. Moreover, the use of miR-145-5p and miR-146b-5p mimic/inhibitor systems before infection of H9C2 cells decreased parasite load, 72 h postinfection. When H9C2 cells were treated with miR-145-5p and miR-146b-5p mimic/inhibitor 48 h after infection, all the used systems, except the miR-146b-5p inhibitor, reduced parasite load. Altogether, our data indicate that these microRNAs putatively control signaling pathways crucial for parasite–host cell interaction. Thus, miR-145-5p and miR-146b-5p deserve to be further investigated as biomarkers of parasite control and tools to identify therapeutic adjuvants to etiological treatment in Chagas disease.

## Introduction

Chagas disease (CD) is an infectious disease caused by the protozoan parasite *Trypanosoma cruzi*. Currently, 6–7 million people are infected and approximately 100 million at risk of infection, placing CD as a major public health problem in Latin America^1,2^. Nevertheless, in the last few decades CD has become a worldwide concern due to human migration and infected individuals from endemic countries are moving to regions in North America and Europe, searching for better quality of life causing a public health problem in countries as United States, France, Italy and Spain^3^. The cardiac form, also called chronic chagasic cardiomyopathy (CCC) is the most frequent and severe form of the disease, and one of the main causes of morbidity and mortality from cardiovascular problems in endemic areas^3^. Developing after decades of indeterminate form, condition without apparent disease, CCC is often triggered by the imbalance between *T. cruzi* and host immune response^4^. Further, CCC is characterized by the establishment of a continuous inflammatory activation due to parasite persistence causing immune-mediated myocardium damage and affecting electrophysiological properties of the heart leading to manifestations as arrhythmias, tissue fibrosis, cardiac hypertrophy, heart failure, and thromboembolism^4–6^.

There are only two drugs currently available for the etiological treatment of CD: Benznidazole (Bz) and Nifurtimox. Benznidazole is a nitroimidazole derivative developed in the early 1970s^6^ and is a prodrug, which exerts its effect after activation by the type I trypanosomal nitro-reductase enzyme, inherent in *T. cruzi* and other protozoa, thus producing reactive metabolites that bind covalently to proteins, lipids, DNA, and RNA resulting in damage to these macromolecules inducing oxidative stress and, consequently, causing death of intra and extracellular forms of the parasite^7,8^. Nifurtimox is a nitrofuran compound that has only recently been approved by the FDA^9^, and is usually selected for treatment when the patient is non-responsive to Bz, showing high rates of associated side effects^6,10^. In addition to its trypanosomicidal effect, Bz also shows some interesting immunomodulatory properties to be completely unveiled^11–15^. Thus, the understanding of Bz mechanism of action and additional effector properties are required. Pentoxifylline (PTX) is a methylxanthine derivative with phosphodiesterase inhibitor activity used to treat intermittent claudication. PTX is known to ameliorate rheological properties of blood mainly by decreasing viscosity, plasma fibrinogen levels, erythrocyte and platelet aggregation^16^. Further, PTX has also shown immunomodulatory and cardioprotective effects, improving ventricular ejection fraction and reducing cardiovascular risk, in clinical trials to treat idiopathic dilated cardiomyopathy^17^ and ischemic cardiomyopathy^18^. We have previously reported that PTX administration to mice with signs of CCC was able to reverse immunological abnormalities and cardiac electrical alterations, and to reduce myocarditis, preventing progression of heart tissue injury^19^. Further, the combined Bz + PTX therapy was able to reduce parasitemia, reverse electrical abnormalities, and most importantly, sustained the beneficial effects in controlling parasite load and reducing electrical abnormalities even after therapy cessation^15^.

MicroRNAs are noncoding single stranded RNA molecules of approximately 22 nucleotides, derived from hairpin structures and loaded into the argonaute protein of the silencing complex, forming imperfect base pairing with 3′-UTR of target sequences, thus repressing translation or promoting degradation of target mRNAs^20,21^. These RNA silencing molecules are usually involved in suppressing gene expression and have been found dysregulated in a variety of diseases, including a broad range of cardiac disorders^22,23^, bacterial^24^, viral^25,26^ and parasite infections^27–30^. In CD, miRNA expression has been studied in heart tissue of transplanted CCC patients^31,32^, in a model of acute Chagas’ heart disease^33,34^ and thymic epithelial cells from *T. cruzi*-infected mice^35^. Additionally, the potential of specific microRNAs to be used as biomarkers for the indeterminate form of CD^36^ and as cardiac remodeling and fibrosis predictors had been investigated^37^.

The microRNAs miR-145-5p and miR-146b-5p have been previously found downregulated and upregulated, respectively, in a model of acute Chagas’ heart disease^33^. In this model, reduced miR-145-5p expression in the cardiac tissue of acutely *T. cruzi*-infected C57BL/6 mice, was directly correlated with parasitemia and prolonged QTc interval, suggesting a critical role for this microRNA in the development of CCC. Further, target filter analysis revealed GJA5 (gap junction protein alpha 5), RNF207 (ring finger protein 7) and KCNA1 (potassium voltage-gated channel shaker-related subfamily) as targets for this microRNA, suggesting an alteration of electrophysiological properties of the heart^33^. Although potentially involved in tumor suppression^38,39^, in the cardiovascular system miR-145 plays a critical regulatory role in vascular smooth muscle cells, actively participating in angiogenesis, and has been found regulated during cardiac remodeling as a response to injury, decreasing levels of vascular endothelial growth factor expression^40^. Moreover, circulating miR-145 has been proposed as a novel biomarker for predicting long-term outcome after acute myocardial infarction^41^.

The miR-146 family comprises two microRNAs, miR-146a-5p and miR-146b-5p, that are highly conserved across species and differ only by two nucleotides on the 3′ end, being first identified in mouse cardiac tissue and later found in humans^42,43^. miR-146b-5p has some identified upstream regulators, i.e. molecules that act as transcription factors, such as NF-κB (nuclear factor kappa-light-chain-enhancer of activated B cells), C/EBPβ (CCAAT/enhancer-binding protein beta) and STAT3/6 (signal transducer and activator of transcription 3/6), all involved in the regulation of expression of genes involved in immune response and inflammatory process^43^. Moreover, it has been shown that miR-146 upregulation is highly dependent on inflammatory stimulus, specially IL-1β, TNF and IFN-γ^44^ all classically found upregulated in Chagas’ heart disease^45–47^. Due to the ambiguity in the seed sequence of miR-146a-5p and miR-146b-5p, there is an obvious limitation on target prediction of these microRNAs, but previous studies identified IRAK1 (interleukin-1 receptor-associated kinase 1) and TRAF6 (tumor necrosis factor receptor-associated factor 6), as key targets of miR-146 family, acting as negative feedback regulators of TLR4 activation cascade^43^. Other study identified TIMP-4, MMP16 and TGIF1 (TGFβ-induced factor homeobox 1) as potential targets involved in the established cardiomyocyte fibrosis^48,49^. In cardiac disorders, miR-146b-5p has been found significantly upregulated in peripheral blood mononuclear cells of patients with coronary artery disease, following remarkedly decrease after atorvastatin and enalapril treatment^50^. Additionally, another study found pronounced miR-146b-5p upregulation in patients with atrial fibrosis and identified TIMP-4 (metalloproteinase inhibitor 4) as potential target for this microRNA, causing its downregulation and, therefore, stimulating the activation of MMP-9 (matrix metallopeptidase 9), resulting in increased collagen synthesis^48^. Moreover, miR-146b-5p was found upregulated in conditions of myocardial ischemia in the plasma of patients, in the infarct zone of murine cardiac tissue, and in several cell subtypes subjected to hypoxia conditions, with fibroblasts and macrophages showing the largest increase in expression, driven by NF-κB stimulation^51^. In CD, miR-146b was previously described as upregulated in the heart tissue of mice acutely infected with the Colombian *T. cruzi* strain^33^.

The data described above support involvement of the mRNAs miR-145-5p and miR-146b-5p in the physiological pathogenesis of cardiac diseases, particularly, in experimental CD. Thus, in the present study, we propose the establishment of a CD in vitro experimental model using the H9C2 cell line of rat cardiomyoblasts infected with the Colombian *T. cruzi* strain to investigate parasite-host interplay, focusing on the effect of parasite infection on the expression of miR-145-5p and miR-146b-5p. Furthermore, we used this model to evaluate the potential of these miRNA as biomarkers of parasite control, evaluating their expression levels under treatment of infected cells with the trypanosomicidal drug Benznidazole (Bz) alone or combined with the immunoregulator Pentoxifylline (PTX).

## Materials and methods

### Cell culture

H9C2 (2-1) rat cardiomyoblast cell line was purchased from Banco de Células do Rio de Janeiro (BCRJ, code: 0098) and cultured in high glucose (4500 mg/L) Dulbecco's Modified Eagle's medium (DMEM) supplemented with pyrogen-free 10% fetal bovine serum (FBS) and 100 μg/mL penicillin/streptomycin under an atmosphere of 5% CO_2_ at 37 °C. VERO cells were cultured in RPMI medium supplemented with HEPES, 5% FBS and 100 μg/mL penicillin/streptomycin under an atmosphere of 5% CO_2_ at 37 °C. Both cell lines were submitted to Mycoplasma detection following a previously stablished protocol^52,53^. All reagents were purchased from ThermoFisher Scientific, Brazil.

### H9C2 cells infection

Trypomastigote forms of *T. cruzi* (Colombian strain, DTU I^54^ were obtained through infection in VERO cells. For that, 10^6^ VERO cells were infected with 10^7^ trypomastigotes for 48 h under an atmosphere of 5% CO_2_ at 37 °C to allow parasite–cell interaction. After 48 h, cells were washed with phosphate-buffered saline (PBS, pH 7.4) and after 6 days the trypomastigotes were collected from the supernatant, submitted to a centrifugation step at 4000×*g* for 10 min to retrieve the parasites following subsequent infection of H9C2 cells. Cover slips were placed on 24 well-plate and treated with Poly-l-Lysin for 2 h, let dry for approximately 1 h and added with 5 × 10^3^ H9C2 cells. For definition of better multiplicity of infection (MOI), 2.5 × 10^4^ (5:1), 5 × 10^4^ (10:1) and 1 × 10^5^ (20:1) of *T. cruzi* trypomastigotes were added in the wells for 4 h to allow parasite–cell interaction. After, cells were washed with PBS pH 7.4 to eliminate non-adhered parasites and collected at 24- and 48-h post-infection washed with PBS, stained with panoptic following manufacturer’s protocol and mounted with Permount (ThermoFisher Scientific, USA). *T. cruzi* infected H9C2 cells and intracellular amastigotes forms/cell were counted (200 cells/coverslip), endocytic index was calculated as % infected cells × amastigotes forms/cells. Additionally, for each H9C2 infection experiment, 5 × 10^4^ H9C2 cells were seeded in 6-well plate and infected with 5 × 10^5^ of *T. cruzi* trypomastigotes (10:1) for 4 h to allow parasite–cell interaction. After, H9C2 cells were washed with PBS pH 7.4 and collected at different time points following specific experimental protocols as shown in the Results section. Representative images were acquired on a light microscope coupled with a digital camera DS-L3 (Nikon Corporation, Sendai, Japan). The experiment was repeated three times, using different cell culture flasks in each experimental condition.

### Benznidazole and PTX treatment

Benznidazole powder was obtained from Laboratório de Farmacotécnica Experimental (Farmanguinhos/FIOCRUZ), being first diluted in DMSO at a concentration of 100 mM, following subsequent dilutions in PBS pH 7.4 at concentrations of 5 mM, 2.5 mM, 1 mM and 0.3 mM prior use at the experiments. Pentoxifylline was commercially purchased as injectable solution under the name of Trental 100 mg/5 mL (Sanofi-Aventis, USA). Dilutions were done in PBS pH 7.4 at concentrations of 20 mg/mL, 10 mg/mL, 5 mg/mL, 2.5 mg/mL and 1.25 mg/mL prior use at the experiments The experiment was repeated three times, using different cell culture flasks in each experimental condition.

### Cellular viability assay

Cellular viability was verified in 1 × 10^3^ cells previously grown in a 96-well plate under an atmosphere of 5% CO_2_ at 37 °C. Cells were treated with DMSO at 0.1%, Bz, PTX and the combined therapy of Bz + PTX. Cells were grown under treatment for 96 h under an atmosphere of 5% CO_2_ at 37 °C and cellular viability was determined using Thiazolyl Blue Tetrazolium Bromide (MTT) assay following protocol previously described^55^. The plates were read at 570 nm using a Molecular Devices SpectraMax Plus microplate reader (California, USA). The experiment was repeated three times, using different cell culture flasks in each experimental condition.

### Transfection with miR-145-5p and miR-146b-5p mimic/inhibitor system

MicroRNAs mirVana miRNA mimic for miR-145-5p (ThermoFisher, USA. Assay ID MC11480) and miR-146b-5p (Assay ID MC10105) and inhibitor systems for miR-145-5p (Assay ID MH11480) and miR-146b-5p (Assay ID MH10105) were used according to manufacturer’s instructions using Lipofectamine RNAiMAX reagent (Invitrogen, ThermoFisher, USA) and Opti-MEM medium. Mimic and inhibitor systems for miR-145-5p and mimic system for miR-146b-5p were used at the final concentration of 10 pmol and inhibitor system for miR-146b-5p was used at the final concentration of 30 pmol in 6-well plates with previously added 5 × 10^4^ H9C2 cells and incubated under an atmosphere of 5% CO_2_ at 37 °C. All controls are added with Lipofectamine RNAiMAX. The experiment was repeated three times, using different cell culture flasks in each experimental condition.

### DNA extraction and *T. cruzi* parasite load quantification by quantitative real-time PCR

Genomic DNA was extracted from H9C2 cells using High Pure PCR Template Preparation Kit (Roche Diagnostics, Indianapolis, IN), following the manufacturer’s instructions. Monolayers of cells were collected with 500 µL of tissue lysis buffer following extensive homogenization. This homogenate was submitted to DNA extraction, according to the manufacture’s recommendations also using the same DNA extraction kit. In both, at the last step of the protocol, DNA was eluted from the silica column in 100 µL of elution buffer, quantified in a Nanodrop ND2000 spectrophotometer and stored at − 20 °C until further analysis. Amplification of *T. cruzi* satellite DNA was done by using the specific primers^56,57^. Cruzi1 (5′-ASTCGGCTGATCGTTTTCGA-3′) and Cruzi2 (5′-AATTCCTCCAAGCAGCGGAT A-3′), both at 750 nM, and the TaqMan probe Cruzi3 (6FAM–CACACACTGGACACCAA–NFQ–MGB) at 50 nM. Standard curves were done with 10^6^ VERO cells spiked with 10^7^ *T. cruzi* trypomastigotes in Tissue Lysis Buffer and extracted making a 1:10 serial dilution of the eluted DNA in TE buffer, ranging from 10^7^ to 1 parasite equivalents. Real-time PCR reactions were carried out on Applied Biosystems ViiA 7 Real-Time PCR system (Thermo Fisher, USA), using the cycling conditions: 50 °C for 2 min, 94 °C for 10 min, followed by 40 cycles at 95 °C and 58 °C for 1 min, where fluorescence was collected at annealing/extension step after each cycle. All samples were run in duplicate, and threshold was set at 0.02. Parasite load was normalized per ng of DNA in each sample.

### Total RNA extraction, reverse transcription, and microRNA gene expression by quantitative real-time PCR

H9C2 cell monolayers, cultivated in 6-well plate as described, were disrupted through homogenization in 500 µL of Tissue Lysis Buffer from the mirVana miRNA Isolation Kit (Life Technologies). Total RNA was extracted using the same kit, according to the manufacturer's recommendations. Total RNA quantification and purity were assessed in a NanoDrop ND2000 (ThermoFisher). Reverse transcription reactions for the small nucleolar RNA U87 and mature microRNAs miR-145-5p and miR-146b-5p were performed with 10 ng of total RNA using TaqMan MicroRNA Reverse Transcription Kit (Applied Biosystems, ThermoFisher Scientific, USA/Cat no. 4366596) and their respective stem-loop primers, following manufacturer’s instructions. RT reactions (15 µL) were run in an Eppendorf Mastercycler thermocycler for at 16 °C for 30 min, 42 °C at 30 min and 85 °C at 5 min. Quantitative real time RT-qPCR was done in a 10 µL reaction containing 5 µL of 2 × TaqMan Universal PCR Master Mix, 0.5 µL of TaqMan probe belonging to either U87 (assay ID 001712), miR-145-5p (assay ID 002278) or miR-146b-5p (assay ID 001097), 2 µL of cDNA and 2.5 µL of RNase-free water. Real-time PCRs were carried out on Applied Biosystems ViiA 7 Real-Time PCR (Thermo Fisher, USA) thermocycler, using the cycling conditions: 10 min at 95 °C, followed by 40 cycles of 15 s at 95 °C and 60 s at 60 °C. Fluorescence was collected after each cycle, at the annealing/extension step. Raw data files were pre-processed using QuantStudio Real-Time PCR Software (Applied Biosystems, USA) with threshold and baseline corrections for each sample and gene expression results were analyzed and Expression Suite v1.0.3 (Applied Biosystems, USA). Threshold was set at 0.02 for all targets and U87 was set as the reference miRNA once it had down constitutive expression across samples (Fig. S1). Target miRNA levels were estimated by the ∆∆Ct method^58,59^, using non-infected samples as calibrators.

### Statistical analysis

All experiments were performed at least in three technical replicates. For miRNA levels analysis by RT-qPCR, Student’s *t* test or Mann–Whitney Rank Sum test was used to analyze the statistical significance of the observed differences (according to the parametric or nonparametric distribution of the values, respectively) with SigmaPlot for Windows version 12.0 (Systat Software, Inc). Results were expressed as means and standard deviations, differences were considered significant if *p* < 0.05.

## Results

### Colombian *T. cruzi* strain-infected H9C2 cells is a viable in vitro experimental model

**Figure 1:**
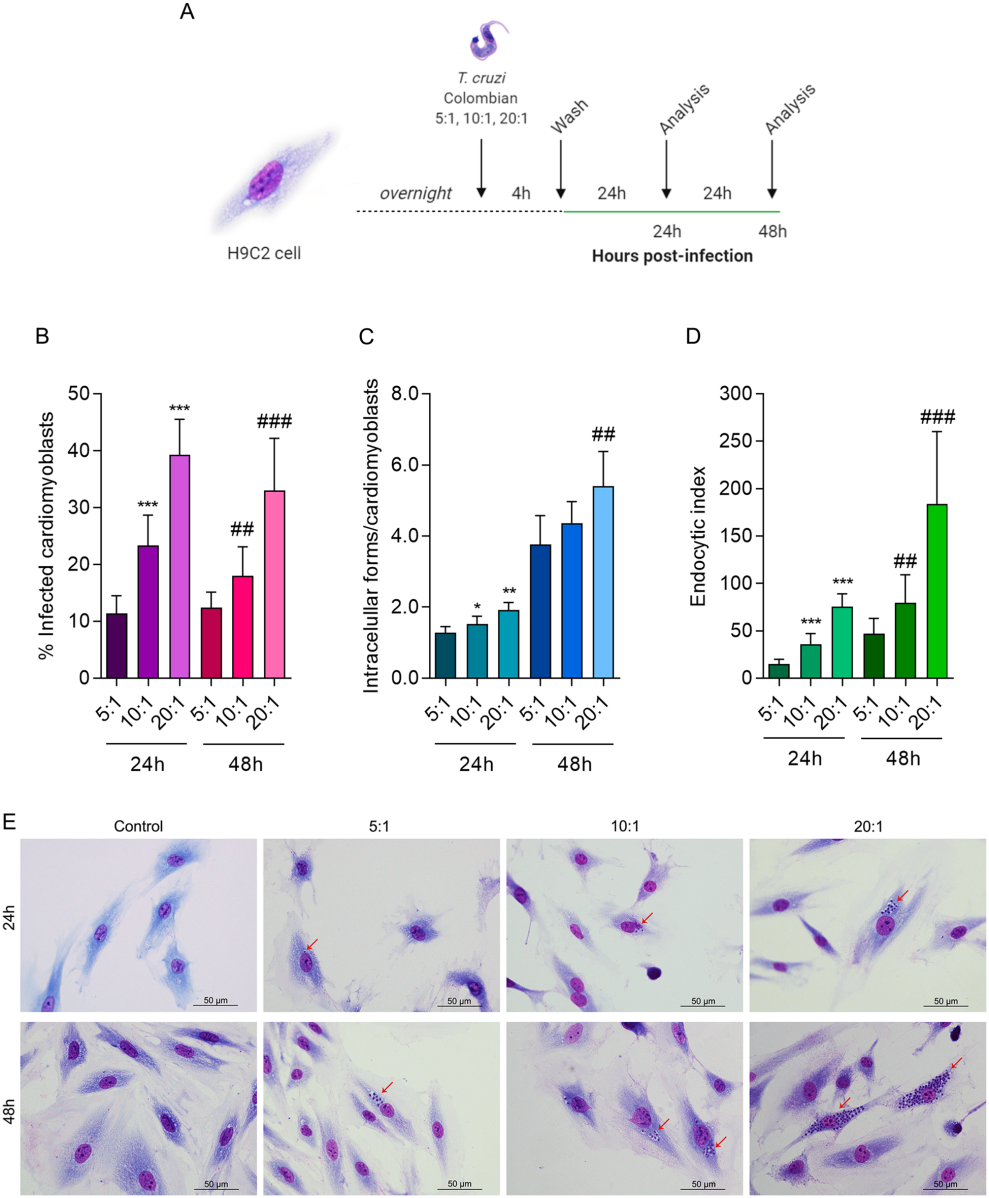
Standardization of *T. cruzi* infection in H9C2 cells. (**A**) H9C2 cells were infected with 5:1, 10:1 and 20:1 *T. cruzi* trypomastigotes for 4 h, washed and collected 24 h and 48 h post-infection. (**B**) Percentage of infected cardiomyoblasts. (**C**) Rate of intracellular forms/cardiomyoblasts. (**D**) Endocytic index. (**E**) Microscopic images of H9C2 cells infected with *T. cruzi* Colombian strain stained with panoptic. Red arrows indicate intracellular forms of *T. cruzi*. For all graphs, significance was determined using unpaired Student’s *t* test: 10:1/24 h, 20:1/24 h vs. 5:1/24 h (*p < 0.05, **p < 0.01, ***p < 0.001); 10:1/48 h, 20:1/484 h vs. 5:1/48 h (^#^p < 0.05, ^##^p < 0.01, ^###^p < 0.001). The experiment was repeated three times, using different cell culture flasks in each experimental condition.

**Figure 2:**
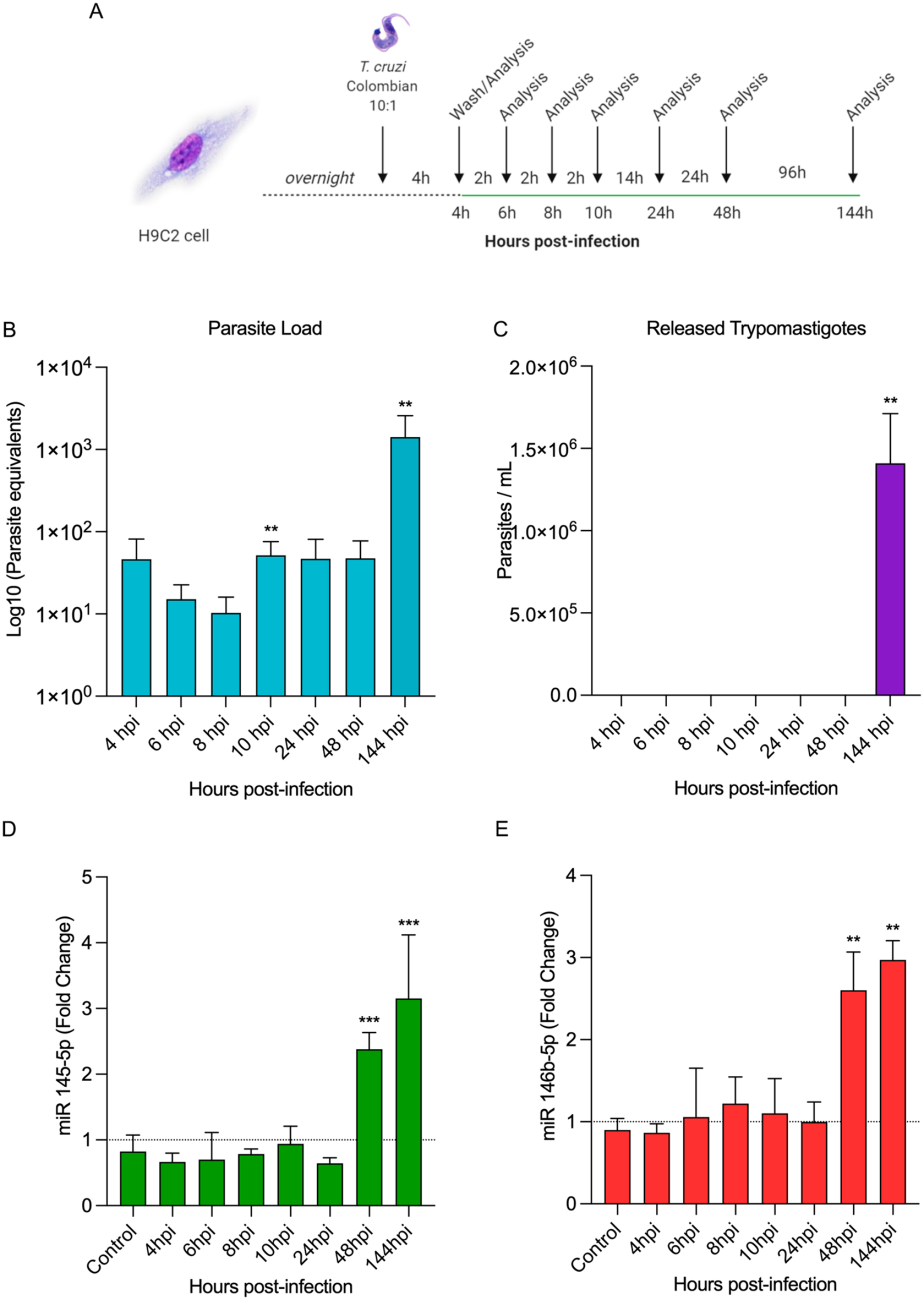
*T. cruzi* infection modulates miR-145-5p and miR-146b-5p expression. (**A**) H9C2 cells were infected with 10:1 *T. cruzi* trypomastigotes for 4 h, washed and collected 4, 6, 8, 10, 24, 48 and 144 h post-infection. (**B**) Parasite load of H9C2 cells infected with *T. cruzi*. (**C**) Concentration of parasites released into the supernatant. (**D**) miR-145-5p levels, (**E**) miR-146b-5p levels. For all graphs, significance was determined using unpaired Student’s *t* test (*p < 0.05, **p < 0.01, ***p < 0.001). The experiment was repeated three times, using different cell culture flasks in each experimental condition.

To evaluate the susceptibility of the H9C2 cell line to infection with the Colombian *T. cruzi* strain, H9C2 cells were infected with trypomastigotes as shown in Fig. 1A. The percentage of infected cells showed significant increase using 10:1 (23.35 ± 5.34) and 20:1 (39.27 ± 6.26), when compared to 5:1, at 24 h postinfection (hpi), and using 10:1 (18.00 ± 5.07) and 20:1 (33.00 ± 9.17) when compared to 5:1 at 48 hpi (Fig. 1B). When counting the number of intracellular forms of *T. cruzi* in the infected cells, it was possible to observe an increase in the number of intracellular forms per cell in a MOI of 10:1 (1.52 ± 0.22) and 20:1 (1.92 ± 0.21), when compared to 5:1 at 24 hpi, and 20:1 (5.40 ± 0.97) but not 10:1 (4.36 ± 0.60), when compared to 5:1 at 48 hpi (Fig. 1C). The endocytic index, showed increased values using a MOI of 10:1 (35.91 ± 11.21) and 20:1 (75.20 ± 13.75), compared to 5:1 at 24 hpi, and a MOI of 10:1 (79.71 ± 29.49) and 20:1 (183.90 ± 76.22), compared to 5:1 at 48 hpi (Fig. 1D). The number of intracellular forms per each cell is easily visualized in the microscopy images, as we detected a proportional amount of amastigote forms inside the cardiomyoblasts as MOIs were increased, at 24 and 48 hpi (Fig. 1E). These results show that H9C2 is a suitable host cell–*T. cruzi* interaction, and to that purpose we have chosen the MOI of 10:1 to proceed with the subsequent experiments. Thus, we evaluated the capability of *T. cruzi* to complete its lifecycle within the H9C2 cell line. For that, we infected H9C2 cells with 10:1 *T. cruzi* trypomastigotes for 4 h to allow parasite–cell interaction and collected samples at 4, 6, 8, 10, 24, 48 and 144 hpi to assess parasite load, released trypomastigotes at supernatant and levels of miR-145-5p and miR-146b-5p levels (Fig. 2A). The kinetic of parasite load inside cells was evaluated at different timepoints, and although parasite load (parasite equivalents) could be detected at 4, 6, 8, 10, 24 and 48 hpi, being significantly increased at 10 hpi and 144 hpi (1413.0 ± 1162.0), compared to the 8 hpi (Fig. 2B). This result shows that at least at 10 hpi the intracellular parasites have not yet undergone into an intense auto-replicative process, which spiked after 144 hpi. The photo documentation of the kinetic of infection shows single intracellular forms at 4, 8, 10, 24 hpi, while amastigote nests were seen at 48 hpi, with significant increase at 144 hpi, when amastigote and trypomastigote forms coexist inside large nests (Fig. S2). Extracellular *T. cruzi* trypomastigote forms were only detected at 144 hpi (1.41 ± 0.30 × 10^6^ trypomastigotes/mL) (Fig. 2C). Therefore, H9C2 cells allowed the complete life cycle of the Colombian *T. cruzi* strain, as described for mammal cells^60^, and showed to be a useful model for in vitro experiments to challenge our questions.

### *Trypanosoma cruzi* infection of H9C2 cells promotes regulation of microRNA miR-145-5p and miR-146b-5p expression

The analysis of microRNA miR-145-5p levels (fold-change) showed no alterations at 4, 6, 8, 10 and 24 hpi but increased levels were detected at 48 (2.38 ± 0.26) and 144 (3.15 ± 0.96) hpi, compared to the controls (Fig. 2D). Additionally, miR-146b-5p levels also presented no alterations at 4, 6, 8, 10 and 24 hpi but showed significant augmentation at 48 (2.60 ± 0.46) and 144 (2.97 ± 0.23) hpi (Fig. 2E). Thus, these data revealed that the upregulation of expression of the miR-145-5p and miR-146b-5p precedes the release of the trypomastigote forms of the parasite from the H9C2 cells, and the highest levels of both microRNAs paralleled the release of the trypomastigote forms.

In order to confirm the specificity of the miR-145-5p and miR-146b-5p TaqMan assays to the miRNAs from H9C2 cells and the absence of cross-reaction with *T. cruzi* total RNA, 10 ng of total RNA, extracted from *T. cruzi* trypomastigotes, was reversed transcribed using the miR-145-5p and miR-146b-5p stem-loop primers, and real time PCRs targeting miR-145-5p and miR-146b-5p were carried out using 2 µL *T. cruzi* cDNA, as described in “Materials and methods”. As reported in Fig. S3, no cross-amplification of *T. cruzi* RNA was detected in the assays, confirming the specificity of the TaqMan assays used herein.

### Combined benznidazole plus pentoxifylline treatment restores miR-145-5p and miR-146b-5p expression levels

**Figure 3:**
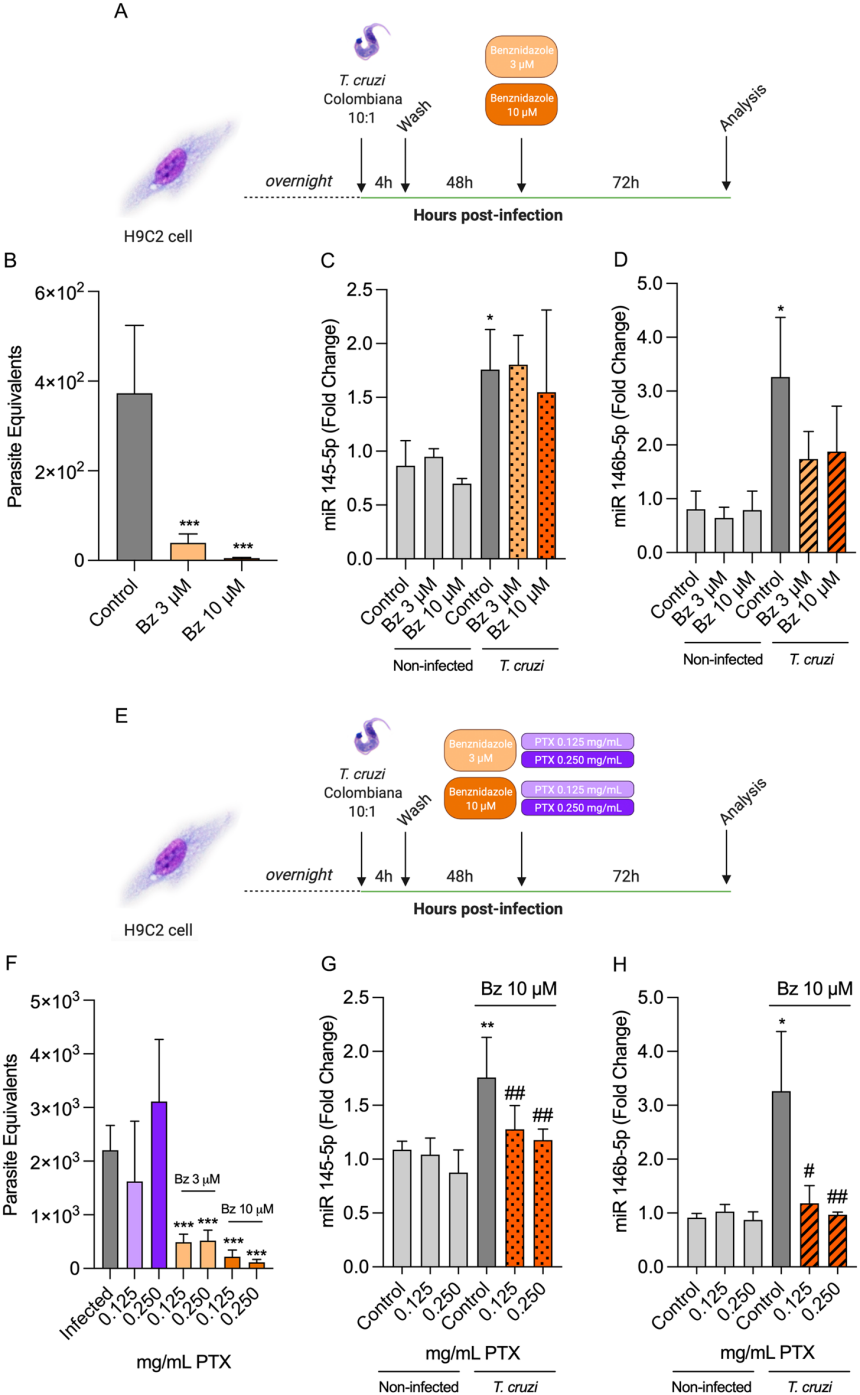
Combined therapy with Benznidazole and Pentoxifylline reverse miR-145-5p and miR-146b-5p upregulation. (**A**) H9C2 cells were infected with 10:1 *T. cruzi* trypomastigotes for 4 h, washed, treated 48 h post-infection with 3 µM and 5 µM of Bz and collected 72 h later. (**B**) Parasite load of H9C2 cells infected with *T. cruzi* and treated with 3 µM and 5 µM of Bz. (**C**) miR-145-5p gene expression, (**D**) miR-146b-5p gene expression, (**E**) H9C2 cells were infected with 10:1 *T. cruzi* trypomastigotes for 4 h, washed, treated 48 h post-infection with 3 µM and 5 µM of Bz + PTX 0.125 mg/mL and 0.250 mg/mL and collected 72 h later. (**F**) Parasite load of H9C2 cells infected with *T. cruzi* and treated with Bz + PTX. (**G**) miR-145-5p levels, (**H**) miR-145-5p levels. For all graphs, significance was determined using unpaired Student’s *t* test or Mann–Whitney Rank Sum (*p < 0.05, **p < 0.01, ***p < 0.001. The experiment was repeated three times, using different cell culture flasks in each experimental condition.

Cells were submitted to MTT assay to test cell viability (% of viable cells) under treatment with Bz at 3 µM (93.69% ± 4.24), 10 µM (84.47% ± 4.50), 25 µM (77.71% ± 10.58), 50 µM (78.68% ± 4.85) and 100 µM (70.58% ± 7.89), compared with untreated (99.83% ± 1.85) and DMSO (99.20% ± 7.85) controls. Thus, the concentration of 3 µM and 10 µM were selected to proceed with experiments mainly because it did not abrogate cell viability as higher Bz concentrations tested (Fig. S4A). Aiming to evaluate if the levels of miR-145-5p and miR-146b-5p could be modulated under etiological treatment, H9C2 cardiomyoblasts were infected, treated and analyzed according to Fig. 3A. Benznidazole was able to decrease parasite load in cells at 3 µM (39.30 ± 19.93 parasite equivalents) and at 10 µM (5.13 ± 1.62 parasite equivalents) compared to the vehicle-treated infected control (372.7 ± 151.8 parasite equivalents) (Fig. 3B). miR-145-5p levels were not altered in non-infected H9C2 cells under Bz treatment (control) but were increased in the *T. cruzi*-infected group (1.76 ± 0.37 times higher than the control group). Similar levels of miR-145-5p were detected after Bz treatment at 3 µM and 10 µM (Fig. 3C). miR-146b-5p levels were unaltered in non-infected H9C2 cell under Bz treatment (control) but were increased in the infected group (3.263 ± 1.11 times higher than the uninfected and untreated cells). The treatment with Bz tended to decrease the miR-146b-5p levels at 3 µM (1.74 ± 0.51) and 10 µM (1.88 ± 0.85), when compared to the infected group (Fig. 3D). As previously proposed for in vivo experiments^61,62^, we evaluated the effect of the immunomodulatory agent Pentoxifylline combined with Bz treatment. The concentrations of 0.125 mg/mL and 0.250 mg/mL did not affect cell viability (Fig. S4B). Next, we tested the cell viability using the combined therapy with Bz 3 µM and Bz 10 µM with increasing concentrations of PTX. The concentrations of Bz 3 µM + PTX 0.125 mg/mL (99.44 ± 11.15% of viable cells), Bz 3 µM + PTX 0.250 mg/mL (90.02 ± 14.03%), Bz 10 µM + PTX 0.125 mg/mL (101.10 ± 16.20%) and Bz 10 µM + PTX 0.250 mg/mL (81.32 ± 10.78%) were chosen to proceed with subsequent assays (Fig. S4C). To evaluate if the expression levels of miR-145-5p and miR-146b-5p were influenced by etiological treatment combined with the immunomodulatory agent, H9C2 cardiomyoblasts were infected, treated and analyzed according to Fig. 3E. Compared with untreated control (2205.00 ± 462.2 parasite equivalents), PTX was not able to control parasite load at both 0.125 mg/mL and 0.250 mg/mL. Nevertheless, Bz 3 µM + 0.125 mg/mL (488.30 ± 151.4 parasite equivalents) and Bz 3 µM + 0.250 mg/mL (517.70 ± 196.9 parasite equivalents) were capable to decrease parasite load, but to a less extent in comparison to Bz 10 µM + 0.125 mg/mL (217.70 ± 125.40 parasite equivalents) and Bz 10 µM + 0.125 mg/mL (116.20 ± 52.01 parasite equivalents) (Fig. 3F). Thus, since Bz 10 µM + PTX 0.125/0.250 mg/mL was able to abrogate parasite replication more efficiently, both of concentrations were selected to evaluate the microRNAs expression. miR-145-5p levels were not altered in non-infected control under PTX treatment but elevated in the *T. cruzi*-infected group (1.76 ± 0.37 times higher than the uninfected control group) and had decreased expression under combined treatment with Bz 10 µM + PTX 0.125 mg/mL (1.28 ± 0.22 times higher than the uninfected control group) and Bz 10 µM + PTX 0.250 mg/mL (1.18 ± 0.10 times higher than the uninfected control group) (Fig. 3G). Additionally, miR-146b-5p level also did not show any alterations in non-infected controls under PTX treatment but were increased in the *T. cruzi*-infected control group (3.263 ± 1.11 times higher than the uninfected control group) and decreased expression under combined treatment with Bz 10 µM + PTX 0.125 mg/mL (1.18 ± 0.33 times higher than the uninfected control group) and Bz 10 µM + PTX 0.250 mg/mL (0.97 ± 0.05 times higher than the uninfected control group) (Fig. 3H). More importantly, treatment with Bz 10 µM + PTX 0.125 mg/mL and Bz 10 µM + PTX 0.250 mg/mL restored expression of miR-146b-5p to basal levels, as there was no significant difference between these groups and the non-infected controls. These results show that the levels of miR-145-5p and miR-146b-5p are increased under *T. cruzi* infection and restored to the same levels of uninfected cells after treatment with combined therapy with Bz + PTX.

### Positive and negative modulation of miR-145-5p and miR-146b-5p in H9C2 cells affects *T. cruzi* replication

**Figure 4:**
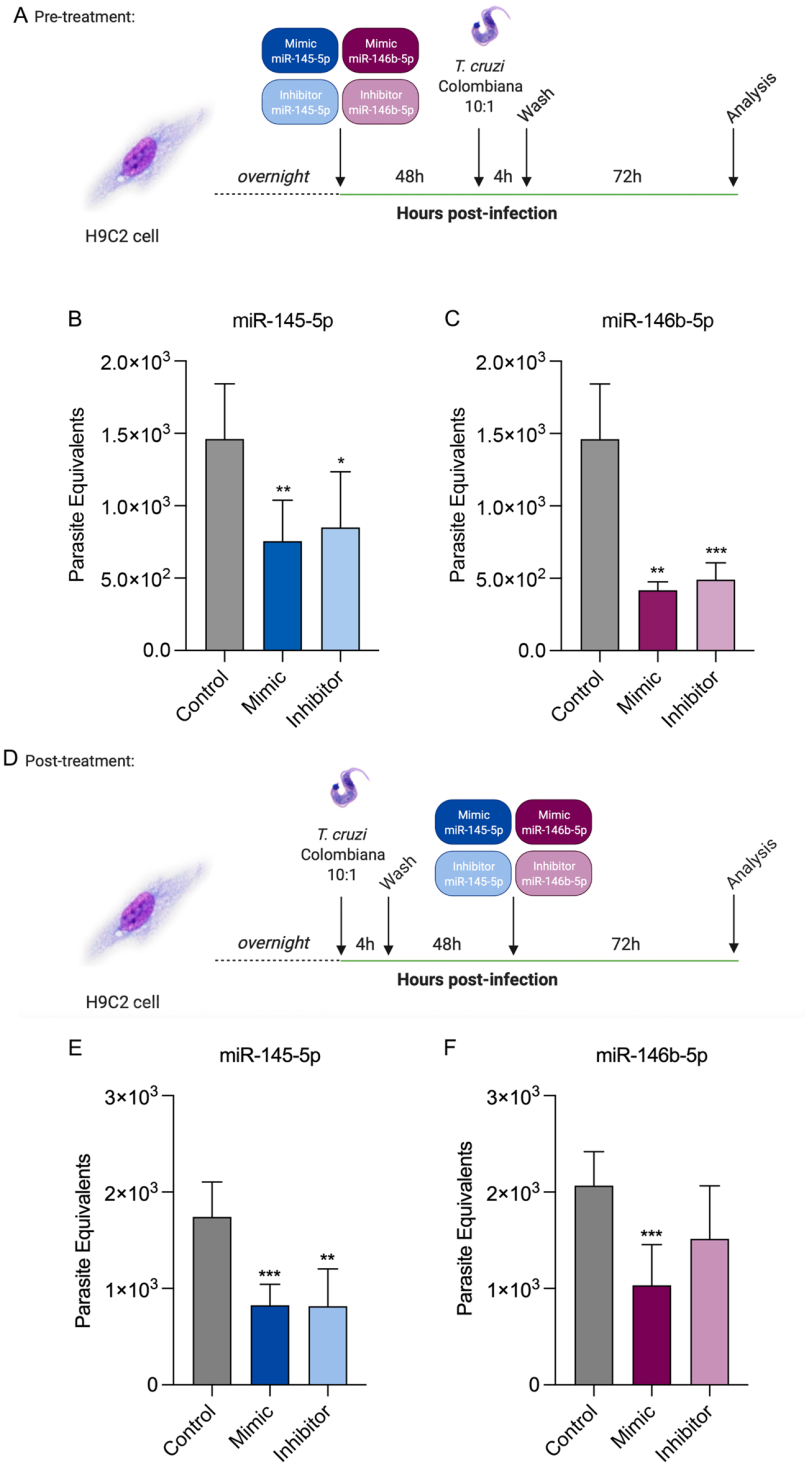
Pre- and post-treatment of miR-145-5p and miR-146b-5p TaqMan^®^ mimic/inhibitor systems alters *T. cruzi* infection dynamics. (**A**) H9C2 cells were pre-treated with miR-145-5p or miR-146b-5p TaqMan mimic/inhibitor systems for 48 h, infected with *T. cruzi* for 4 h, washed and collected 72 h later. (**B**) Parasite load of H9C2 cells pre-treated with miR-145-5p TaqMan mimic/inhibitor system. (**C**) Parasite load of H9C2 cells pre-treated with miR-146b-5p TaqMan mimic/inhibitor system. (**D**) H9C2 cells were infected with *T. cruzi* for 4 h, washed, post-treated with miR-145-5p or miR-146b-5p TaqMan mimic/inhibitor systems for 48 h and collected 72 h later. (**E**) Parasite load of H9C2 cells post-treated with miR-145-5p TaqMan mimic/inhibitor system. (**F**) Parasite load of H9C2 cells post-treated with miR-146b-5p TaqMan mimic/inhibitor system. All controls are added with Lipofectamine RNAiMAX. For all graphs, significance was determined using unpaired Student’s *t* test or Mann–Whitney Rank Sum (*p < 0.05, **p < 0.01, ***p < 0.001). The experiment was repeated three times, using different cell culture flasks in each experimental condition.

After observing the upregulation of miR-145-5p and miR-146b-5p levels in *T. cruzi*-infected H9C2 cells, we evaluated the effects of up- and down-regulation of these miRNAs in the in vitro experimental infection using a TaqMan miRNA mimic/inhibitor transfection system. First, we assessed the transfection protocol effect on cell viability adding 5, 10 and 30 pmol of miR-145-5p and miR-146b-5p mimic/inhibitor system and collecting cells 96 h hours later. For miR-145-5p, only at concentrations of 10 (84.32 ± 2.39% of viable cells) and 30 pmol (83.68 ± 9.99% of viable cells) of the mimic system a decreasing in cell viability was observed, compared to the control (Fig. S5A). Regarding miR-146b-5p, none of the concentrations used for both mimic and inhibitor systems were capable of decreasing cell viability (Fig. S5B). Therefore, the concentration of 5 pmol was chosen for subsequent experiments, except for the inhibitor system of miR-146b-5p, where the concentration of 30 pmol was chosen, once 5 pmol and 10 pmol was not effective in decreasing gene expression, as this microRNA already has a low basal level in H9C2 cells. Next, H9C2 cells were transfected with miR-145-5p and miR-146b-5p mimic/inhibitor systems (Fig. S5C). miR-145-5p mimic system successfully increased its expression at both 24 (16,661 ± 1166 times higher than non-transfected cells) and 48 h (34,182 ± 3910 times higher than non-transfected cells) compared to control (Fig. S5D). Additionally, miR-145-5p inhibitor system also showed efficient transfection decreasing gene expression at 24 (0.14 ± 0.02 times lower than non-transfected cells) and 48 (0.20 ± 0.04 times lower than non-transfected cells) hours, compared to controls (Fig. S5E). miR-146b-5p mimic system worked efficiently at 24 (166.60 ± 11.66 times higher than non-transfected cells) and 48 h (341.80 ± 39.10 times higher than non-transfected cells) compared to the control (Fig S5F), and the inhibitor system also promoted decreasing in gene expression at 24 h (0.51 ± 0.13 times lower than non-transfected cells) compared to controls (Fig. S5G). Subsequently, we evaluated the effect of transfecting cells prior to infection in parasite load according to Fig. 4A. MicroRNA miR-145-5p modulation resulted in a decrease in parasite load in both mimic (755.70 parasite equivalents ± 282.20) and inhibitor (850.30 parasite equivalents ± 384.50) systems compared to control (1461 parasite equivalents ± 380.90) (Fig. 4B). Modulation of miR-146b-5p also resulted in a decrease in parasite load in both mimic (417.20 ± 58.12 parasite equivalents) and inhibitor (489.80 ± 117.50 parasite equivalents) systems compared to control (1461 ± 380.90 parasite equivalents) (Fig. 4C). Next, we evaluated the effects of transfecting cells after *T. cruzi* infection according to Fig. 4D. MicroRNA miR-145-5p modulation resulted in a decrease in parasite load in both mimic (824.70 ± 217.20 parasite equivalents) and inhibitor (813.30 ± 386.60 parasite equivalents) systems compared to controls (1743 ± 360.30 parasite equivalents) (Fig. 4E), and miR-146b-5p modulation also resulted in a significant decrease in parasite load in mimic (1032 ± 422.6 parasite equivalents) but not inhibitor (1515 ± 548.50 parasite equivalents) systems compared to controls (2067 ± 352.20 parasite equivalents) (Fig. 4F). These results come as an interesting finding, showing that positive and negative modulation of microRNAs prior and after *T. cruzi* infection may alter the parasite cycle dynamics within the cell, promoting decreasing in parasite load.

## Discussion

Chronic chagasic cardiomyopathy is the most frequent and severe form of CD, being the main cause of morbidity by cardiovascular impairment in endemic areas^63^. Efforts have been made to understand the role of global alterations of microRNAs expression profiles in controlling CCC pathogenesis related pathways^33,34^ and previous studies focused on specific microRNA profiles^32,64^. The cardiac tissue is an important site for *T. cruzi* persistence in the host during the chronic phase of infection^65^, therefore, in this study, firstly we propose the validation of an in vitro experimental model to investigate *T. cruzi* interactions with the host cell using the rat H9C2 cardiomyoblast cell line, which will spare the use of animals to obtain primary cardiomyocyte cultures and speed off the investigation of parasite–host interactions. The Colombian *T. cruzi* strain infection of H9C2 cells revealed increased intracellular parasite load and release of trypomastigote forms at 144 hpi, suggesting a successful infection and the complete parasite cycle, as expected to be detected in a mammal cell^60^. Although the Colombian *T. cruzi* strain is knowingly recognized as cardiotropic, it was also able to successfully infect and complete the parasite cycle in astrocytes, the main parasite auberge in the central nervous system^66,67^. Altogether, our results suggest that the H9C2 cardiomyocyte cell line is capable of sustaining the lifecycle of the Colombian *T. cruzi* strain and, therefore, is a suitable cell for in vitro experimental model of CD to explore host–parasite interaction. Additionally, primary cardiomyocytes and H9C2 show similar hypertrophic responses, and might be interchangeable for prospective molecular studies in heart development and diseases^68^.

Amastigote forms of *T. cruzi* started intracellular development after 24 hpi and showed increase of numbers at 48 hpi, meaning they are going through intense division, suggesting that *T. cruzi* replication and releasing might be causing, directly or indirectly, upregulation of these microRNAs. It is well known that during the *T. cruzi*–cardiomyocyte interaction, the parasite start controlling overall host gene expression, including immune response genes, inflammation, cytoskeletal organization among other features^69^, therefore, it is most likely that *T. cruzi* also takes over microRNAs expression. Potential targets for the miR-145 family present in cardiovascular cells include SMAD3, a member of the SMAD family that acts as mediator of the signaling pathway triggered by TGF-β, and PAK1 and PAK4, molecules that play a role in cytoskeleton remodeling, affecting different cellular processes as directional motility, invasion, metastasis, and growth^39^. Thus, the upregulated miR-145-5p might act downregulating genes related to cell organization and growth processes affected by *T. cruzi* infection. A notable result is the increasing levels of miR-145-5p and miR-146b-5p in infected H9C2 cells at 48 hpi, observed before trypomastigotes releasing at 144 hpi. These findings led us to propose that this event should be explored in an in vivo model of chronic CD reactivation. If it is shown to be true, the use of the miR-145-5p and miR-146b-5p miRNAs as surrogate biomarkers could be potentially useful in patients subjected to immunosuppression after heart transplantation, as cardiac biopsies are taken in regular intervals to assess reinfection and could be an important tool to detect those miRNAs even before the increasing of the parasite load in the heart and blood^70^, allowing health professionals to make proper interventions in time to prevent reactivation and its consequences.

The infection caused by *T. cruzi* elicits an immune response that is driven by pro-inflammatory cytokines such as IFN-γ and TNF, chemokines and enzymes and has been shown in several studies that etiological treatment contributes to parasite load reduction and rearrangement of the dysregulated immune response in patients^71,72^ and experimental models^15,71^. Chagas disease cardiomyopathy is a knowingly immune dysregulated disorder^46^, and recent study by our group, using C57BL/6 mice chronically infected with the same Colombian *T. cruzi* strain used in the present study, showed global immune dysregulation with upregulation of key CD cytokines such as IFN-γ, CSF2, IL-12, IL-2 and chemokines such as CCR4, CCL3 and CCL5^73^ corroborating that infection with *T. cruzi* is in fact causing increased production of inflammatory mediators that could be triggering the upregulation of miR-145-5p and, especially miR-146b-5p that is highly dependent of inflammatory stimulus^44^. Besides its trypanosomicidal activity, Bz has also been explored as having anti-inflammatory properties^14^. In a model of primary cardiomyocyte culture infected with the RA *T. cruzi* strain, treatment with suboptimal doses has shown to decrease NOS2 expression, IL-1β and IL-6 production^11^. Additionally, recent study from our group on a 30-day treatment with Bz in the C57BL/6 *T. cruzi* chronic model, showed downregulation of altered pro-inflammatory molecules as CSF2, IL-7 and IL-12, also substantially decreasing the production of IFN-γ and IL-2^73^, which was not sufficient however, to reverse the upregulation of both miRNAs triggered by the infection, although it was effective in controlling parasite replication. A previous study of microRNA transcriptome profiling found miR-146b-5p to be upregulated and associated with parasitemia levels and electrical abnormalities in a model of acute Chagas’ heart disease induced by infection with the Colombian strain^33^, corroborating our data and supporting the importance of therapeutic intervention aiming to decrease the expression of this miRNA. Pentoxifylline is a hemorheological agent that has described anti-inflammatory and antioxidant effects^16^, however, it has no trypanosomicidal effect, meaning that the failure to decrease parasite load showed here was expected and previously described in in vivo model of infection^19^. Moreover, PTX has been studied as an immunomodulatory agent complementary to etiological treatment in CD^15^ and in other parasitic infectious diseases such as cutaneous^74,75^ and mucosal^76,77^ leishmaniasis. Nevertheless, the combined therapy was effective in reversing the upregulation of both miR-145-5p and miR-146b-5p. Additionally to its classically described mechanism of action, PTX can also inhibit a wide range of cytotoxic responses mainly by acting on cytokine production dynamics^78^, downregulating proinflammatory cytokines IFN-γ, GM-CSF and TNF^79^, attenuating cell surface expression of IL-2 receptor and production of IL-8 and CCL2 in pulmonary epithelial cells^80^. Recent study from our group on a 30-day treatment with Bz + PTX in the C57BL/6 *T. cruzi* chronic model, showed regulation of upregulated pro-inflammatory molecules as CSF2, IL-7 and IL-12, also substantially decreasing the production of IFN-γ, CSF2, IL-2 among other immunological molecules^73^ suggesting that the immunomodulation caused by the combined Bz + PTX therapy might be affecting the upregulation of these two miRNAs. It is most likely that the combined Bz + PTX therapy was able to reverse miR-145-5p and miR-146-5p overexpression by combining the trypanosomicidal effect of Bz, successfully eliminating *T. cruzi* and, putatively, reducing the tissue damage caused by the parasites, as well as the immunomodulatory effect of PTX, preventing potential overproduction of cytokines that could culminate in the modulation of potential miRNA targets, that remains to be explored in the present experimental *T. cruzi* model.

Previously, miR-145 has been explored in cancer research, and cardiac system, mainly focusing on myocardial infarction^81–87^. Study using an in vitro model of hypoxia in H9C2 cells, showed upregulation of miR-145 associated with decrease in cell viability and apoptosis, revealing Rac1 (Rac family small GTPase 1) as a target and showing the involvement of PI3K/Akt and MAPK/ERK signaling pathways^82^, which was further confirmed by in vivo experiments^86^. In *T. cruzi*, actin-regulating molecules such as Rac1 CDc42 have been implicated in amastigote invasion, mediating actin recruitment and enhancing invasiveness^88^. It is known that miR-146b-5p is dependent on inflammatory stimulus of specific cytokines such as IFN-γ, TNF and IL-1β^44^, that are also dysregulated in CD^5^, as showed in recent study by our group in cardiac tissue using an experimental model of C57BL/6 mice chronically infected with the Colombian *T. cruzi* strain^73^. In murine *T. cruzi* infection, PTX was able to reverse CCC clinical signs, hampering the progression of heart injury, as improves connexin 43 expression and decreases fibronectin overdeposition, reducing CD8^+^ T-cells expressing activation and migration markers, and of activated blood vessel endothelial cells, and decreased the number of perforin-expressing cells invading the cardiac tissue^19^. Recently, Bz was shown to reduce of IL-6 and TNF production by *T. cruzi*-infected cardiac spheroids^12^ and promote down-regulation of NF-kB by LPS-stimulated cardiomyocytes^11^. Therefore, all these results reinforce the importance of proposing a combined etiological and immunomodulatory treatment for CCC.

The up and downmodulation of miR-145-5p and miR-146b-5p using TaqMan mimic/inhibitor systems as a pre-treatment before *T. cruzi* infection and as a post-treatment after *T. cruzi* infection to evaluate if the miRNA modulation would affect parasite load in H9C2 cardiomyoblast cells. Study on *T. cruzi* infection of Schwann cells revealed that trans-sialidase triggers the survival of host cell via the PI3K/Akt pathway through activation of PI3K, helping the establishment of a successful infection stimulating host antiapoptotic mechanisms^89^. Additionally, a more recent study showed that extracellular amastigotes of *T. cruzi* can activate Akt and ERK molecules, suggesting the participation of both PI3K/Akt and MAPK/ERK signaling pathways, interfering with cytoskeleton rearrangement and phagocytosis, probably subverting the phagocytic machinery allowing a successful infection^90^. It is also known that miR-145-5p specifically affects these pathways^82^. In *T. cruzi*, Rac1 has been previously elucidated as the molecule modulating actin recruitment and enhancing amastigote invasiveness^88,91^. Moreover, Rac1 is a known target of miR-145-5p^82^, thus it is possible that pre- and pos-treatment of cells with miR-145-5p mimic system might be downregulating molecules of this pathway, therefore hampering parasite–host interaction dynamics and impairing the establishment of a successful infection. However, the role played by pre- and post- treatment with miR-145-5p inhibitor system also promoting decrease in parasite load deserves further investigation, once it might be activating signaling pathways that are yet unknown, providing disadvantage to the parasite in the infection. Moreover, pre- and post-treatment with miR-146b-5p mimic/inhibitor systems was also able to significantly decrease infection, except from the post-treatment inhibitor system, which silencing through TaqMan inhibitor system was not as effective in silencing the expression of miR-146b-5p, suggesting the lack of efficiency in reducing parasite load. Treatment with miR-146b-5p mimic might contribute to the downregulation of known targets such as IRAK1 and TRAF6, involved in the TLR4 signaling pathway, and TIMP-4, MMP16 and TGIF1^48,49^. However, pre-treatment with miR-146b-5p inhibitor system seems more prominent once the downregulation of this microRNA might cause the upregulation of these targets. It has been recently discovered that the use of a TLR4 agonist promotes higher survival rate and decreases parasite burdens in BALB/c mice acutely infected with *T. cruzi*, although cardiac damage was not prevented^92^, thus shedding light on how miR-146b-5p is hampering infection of cells and decreasing parasite load. Additionally, silencing of miR-146b-5p in a murine and porcine model of myocardial infarction reduced fibrosis and cell death, restoring cardiac remodeling and heart function^51^, which shows us additional advantages to the further investigation of the use of miR-146b-5p for the treatment of CCC, as well as a tool for identification of putative pathways to be targeted by new therapeutic approaches.

There are several other miRNAs involved in CD establishment and progression^31–35,37,64^. Nevertheless, we evaluated the modulation of two miRNAs based on their regulation in experimental acute CD in previous studies^34,93^, and the involvement of miR-145-5p in cardiomyopathies^82,83,86,94–96^ and miR-146b-5p in inflammatory processes^97,98^ that are known to be dysregulated in CD. The evaluation of only two miRNAs and the absence of the expression analysis of other host mRNAs that can be related to the infection control are a limitation of this study. Additionally, the evaluation of specific time points in between 48 and 144 h in the time-course infection experiment are missing, once we thought it would be more relevant to evaluate the modulation of both miRNAs in early stages of the infection when *T. cruzi* trypomastigotes are in the process of entering the host cell, and in late time points when the infection is already established and trypomastigotes are being actively released from cardiomyocytes, crucial biological processes to support parasite persistence in the heart tissue^69,99,100^. Nevertheless, the results observed here are clear, opening a venue to the search of these miRNAs as new biomarker candidates to monitor the etiological treatment in CD, especially for prognosis prediction and therapeutic cure in the chronic phase of CD^101^. The gold standard assessment for treatment efficacy is still seroconversion of serological tests, which may take decades to become negative^72,101^. In this context, microRNAs could emerge as potential biomarkers mainly because they are present in the circulation and have stable detection, due to several mechanisms that prevent their degradation^102^. miR-1, for example, was firstly reported as a key regulator of cardiac differentiation^103^ and was later validated as potential biomarker in heart failure onset after myocardial infarction, as it showed to negatively correlate with ejection fraction on 49 patients tested^104^. In Chagas disease, miR-208a, a heart specific miRNA that plays a critical role in cardiac dysfunction leading to heart failure, showed increased circulating levels during the chronic indeterminate phase, suggesting that this microRNA could be a potential risk-prediction score biomarker for CCC^36^. Another study investigated six prominent miRNAs involved on cardiac remodeling, cardiac hypertrophy and fibrosis, observed differential expression for miR-19a, miR-21-5p and miR-29b-3p in patients with CCC when compared to indeterminate form patients, that positively correlated with cardiac dysfunction and fibrosis and negatively correlated with ejection fraction, suggesting the potential of these microRNAs as biomarkers of CCC progression^37^. Since it is well known that one single miRNA may target many mRNA transcripts regulating several pathways depending on the condition^20,105^, the participation of miR-145-5p and miR-146b-5p in pathophysiological processes of *T. cruzi* infection might be more relevant than previously thought. It is clear from the results we came across in this study the participation of miR-145-5p and miR-146b-5p in the parasite–host interaction and establishment of *T. cruzi* infection. Additionally, pharmacotherapy being able to modulate these microRNAs might serve as a first insight on the potential role of microRNAs as biomarkers of treatment efficacy, although further validation is needed. Furthermore, the potential use of miR-145-5p and miR-146b-5p as therapeutic adjuvants in the treatment of CD also deserves special attention once they might be acting on the parasite entrance in host cells and could improve traditional Bz treatment efficacy.

## Supplementary Information

Supplementary Legends.

Supplementary Figure 1.

Supplementary Figure 2.

Supplementary Figure 3.

Supplementary Figure 4.

Supplementary Figure 5.
