# Supplementary material for: Modulation of miR-145-5p and miR-146b-5p levels is linked to reduced parasite load in H9C2 Trypanosoma cruzi infected cardiomyoblasts
Source: Sci Rep. 2022 Jan 26;12:1436. doi: 10.1038/s41598-022-05493-4 (PMC8791985; doi:10.1038/s41598-022-05493-4)
Supplement: Supplementary file 1 — Supplementary Legends. [file 41598_2022_5493_MOESM1_ESM.docx]

**Legends to supplementary figures:**

**Supplementary Figure 1. Constitutive expression for U87 reference miRNA candidate in H9C2 cells infected with *T. cruzi*.** The representative graph shows the Ct values (red squares) for RT-qPCR targeting U87 in H9C2 cells infected with *T. cruzi* from 4 to 48 hours post-infection. The experiment was repeated three times, using different cell culture flasks in each experimental condition.

**Supplementary Figure 2. *Trypanosoma* *cruzi* cell cycle in H9C2 cells.** Cells were infected and photographed at 20x magnification in cell culture flasks at 4 hpi, 6 hpi, 8 hpi, 10 hpi, 24 hpi, 48 hpi and 144 hpi. Red arrows indicate intracellular forms of the parasite. The experiment was repeated three times, using different cell culture flasks in each experimental condition.

**Supplementary Figure 3. Specificity analysis of miR-145-5p and miR-146-5p to the RNA from H9C2 cells.** The cross-reaction between the *T. cruzi* RNA (from Trypomastigotes) and the miR-145-5p and miR-146-5p TaqMan assays was investigated. (A) Amplification plots of the RT-qPCR assays targeting miR-145-5p. (B) Amplification plots of the RT-qPCR assays targeting miR-146-5p. The positive controls (H9C2 cells) and negative controls (-RT (reverse transcriptase) control and Negative Template control (NTC)) are showed in both graphs. The experiment was performed with trypomastigotes obtained from 3 independent infected cells culture.

**Supplementary Figure 4. Cell viability assay.** MTT assays for H9C2 cells treated with (A) Benznidazole, (B) Pentoxifylline and (C) Benznidazole and Pentoxiffyline. For all graphs, significance was determined using unpaired Student’s t-test (* p < 0.05, ** p < 0.01, *** p < 0.001). The experiment was repeated three times, using different cell culture flasks in each experimental condition.

**Supplementary Figure 5. Validation of the transfection model using miR-145-5p and miR-146b-5p TaqMan mimic/inhibitor systems.** MTT assay for (A) miR-145-5p and (B) miR-146b-5p. (C) H9C2 cells were transfected with miR-145-5p and miR-146b-5p TaqMan mimic/inhibitor systems and washed and collected 24 and 48h after transfection. (D) Mimic and (E) Inhibitor system for miR-145-5p. (F) Mimic and (G) Inhibitor system for miR-146b-5p. All controls are added with Lipofectamine RNAiMAX. For all graphs, significance was determined using unpaired Student’s t-test (* p < 0.05, ** p < 0.01, *** p < 0.001). The experiment was repeated three times, using different cell culture flasks in each experimental condition.
