# Supplementary figures and images for: Modulation of miR-145-5p and miR-146b-5p levels is linked to reduced parasite load in H9C2 Trypanosoma cruzi infected cardiomyoblasts
Source: Sci Rep. 2022 Jan 26;12:1436. doi: 10.1038/s41598-022-05493-4 (PMC8791985; doi:10.1038/s41598-022-05493-4)

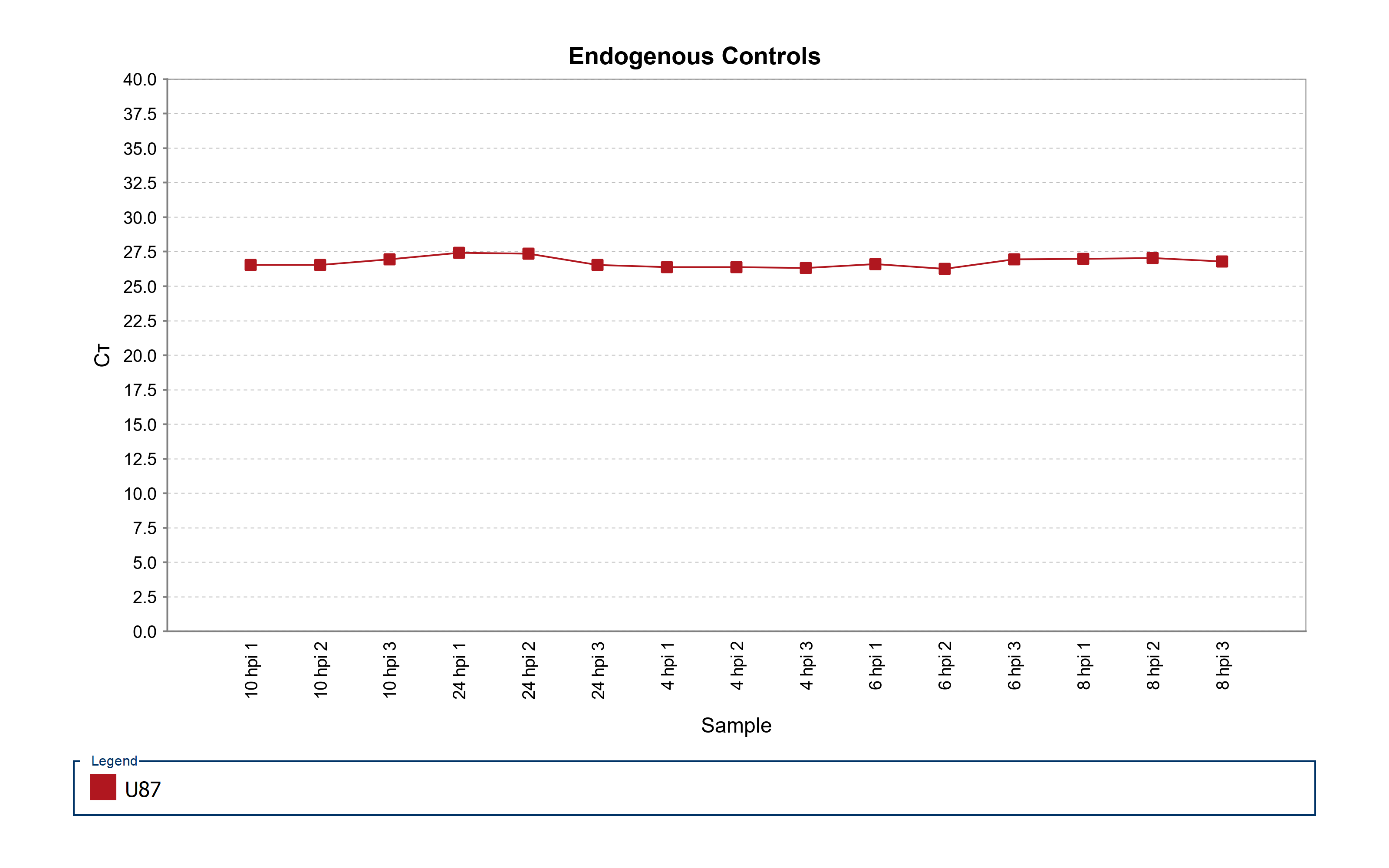

Supplement: Supplementary file 2 — Supplementary Figure 1. [file 41598_2022_5493_MOESM2_ESM.tif]

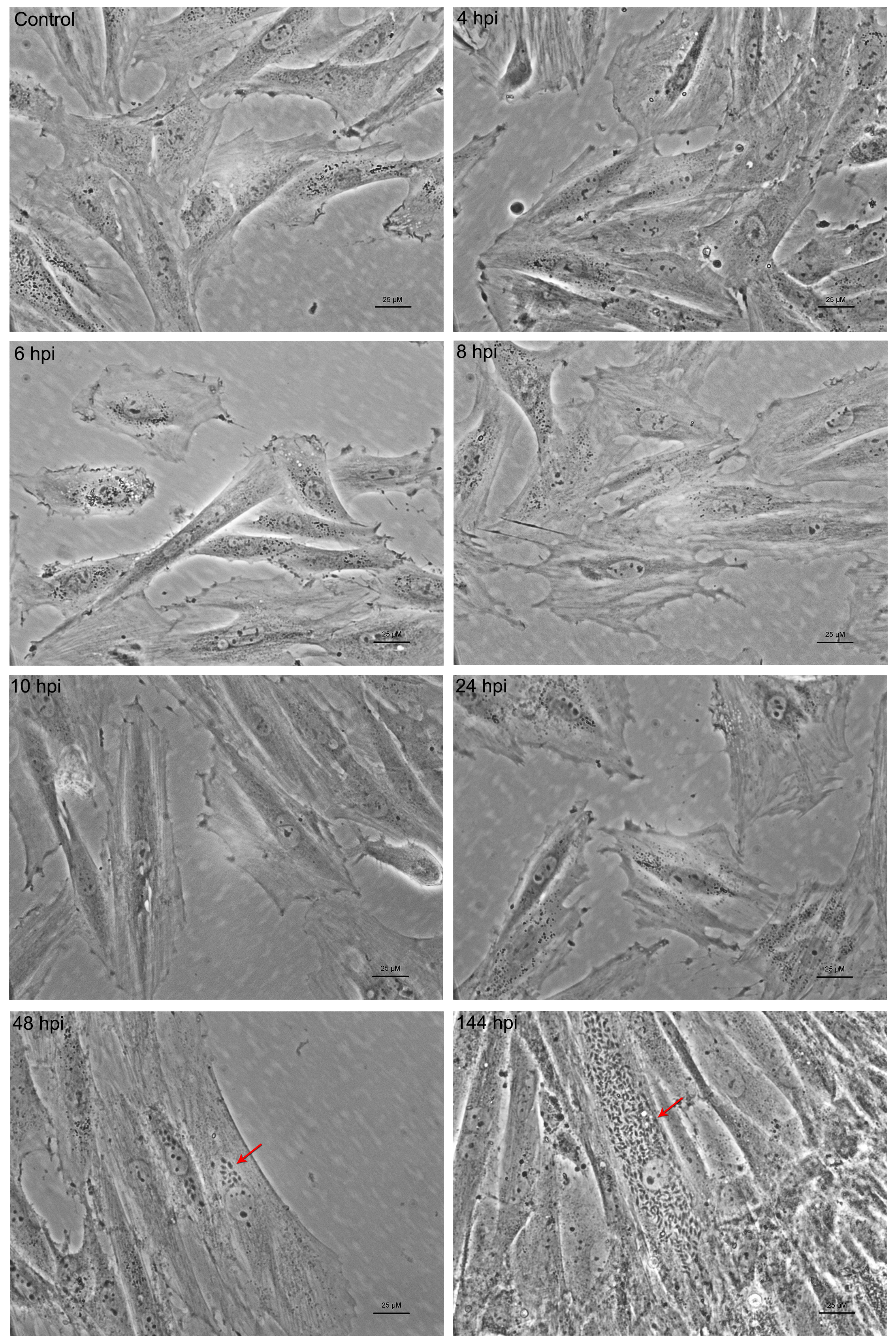

Supplement: Supplementary file 3 — Supplementary Figure 2. [file 41598_2022_5493_MOESM3_ESM.tif]

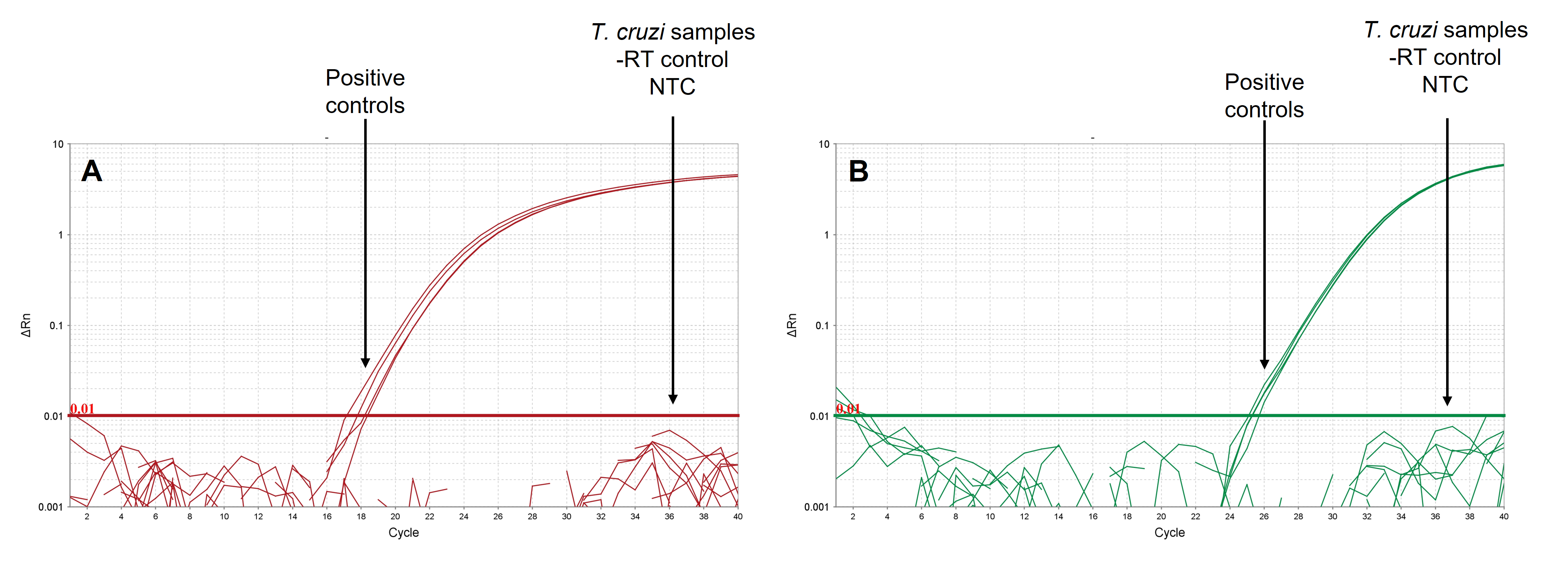

Supplement: Supplementary file 4 — Supplementary Figure 3. [file 41598_2022_5493_MOESM4_ESM.tif]

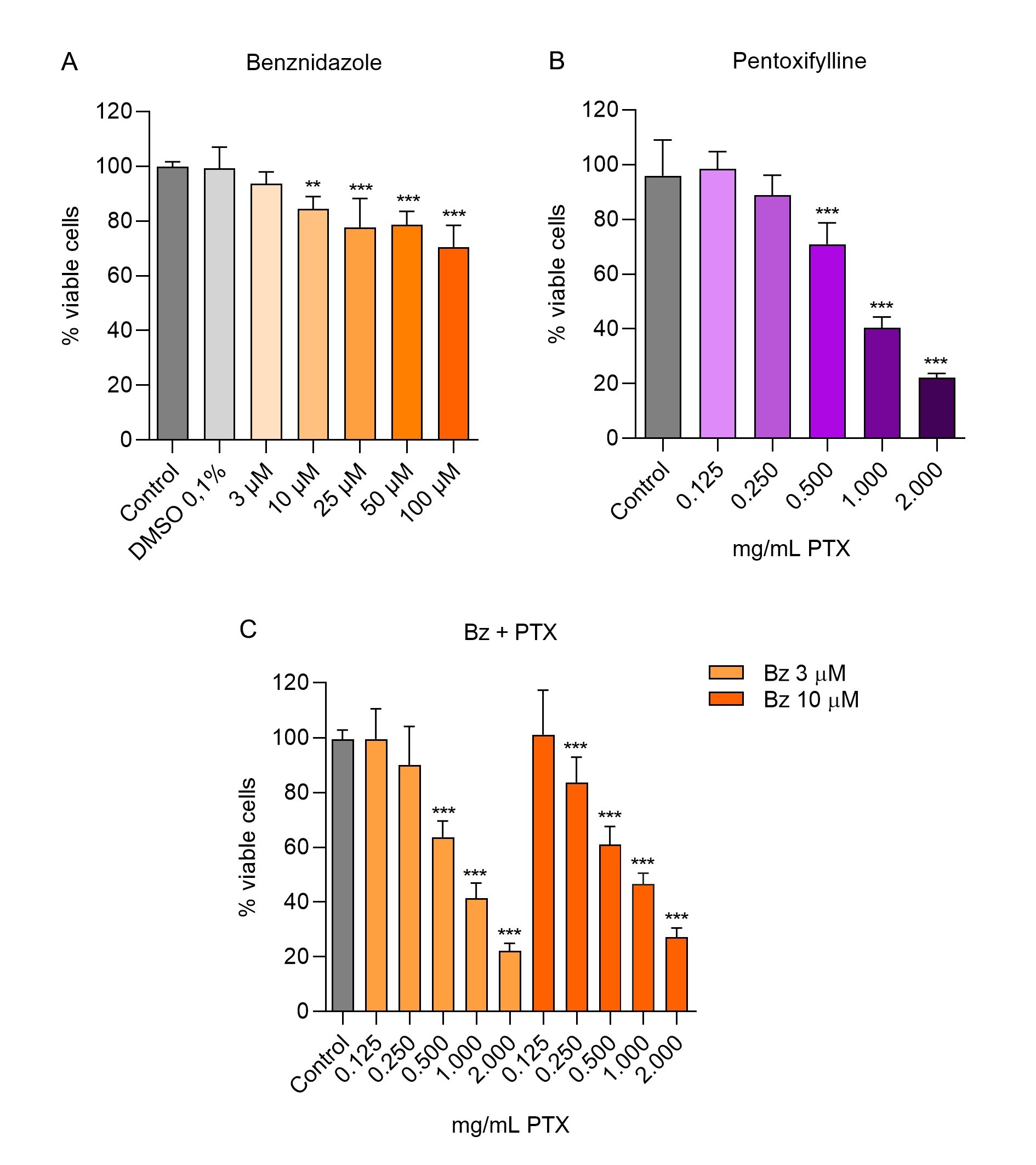

Supplement: Supplementary file 5 — Supplementary Figure 4. [file 41598_2022_5493_MOESM5_ESM.tif]

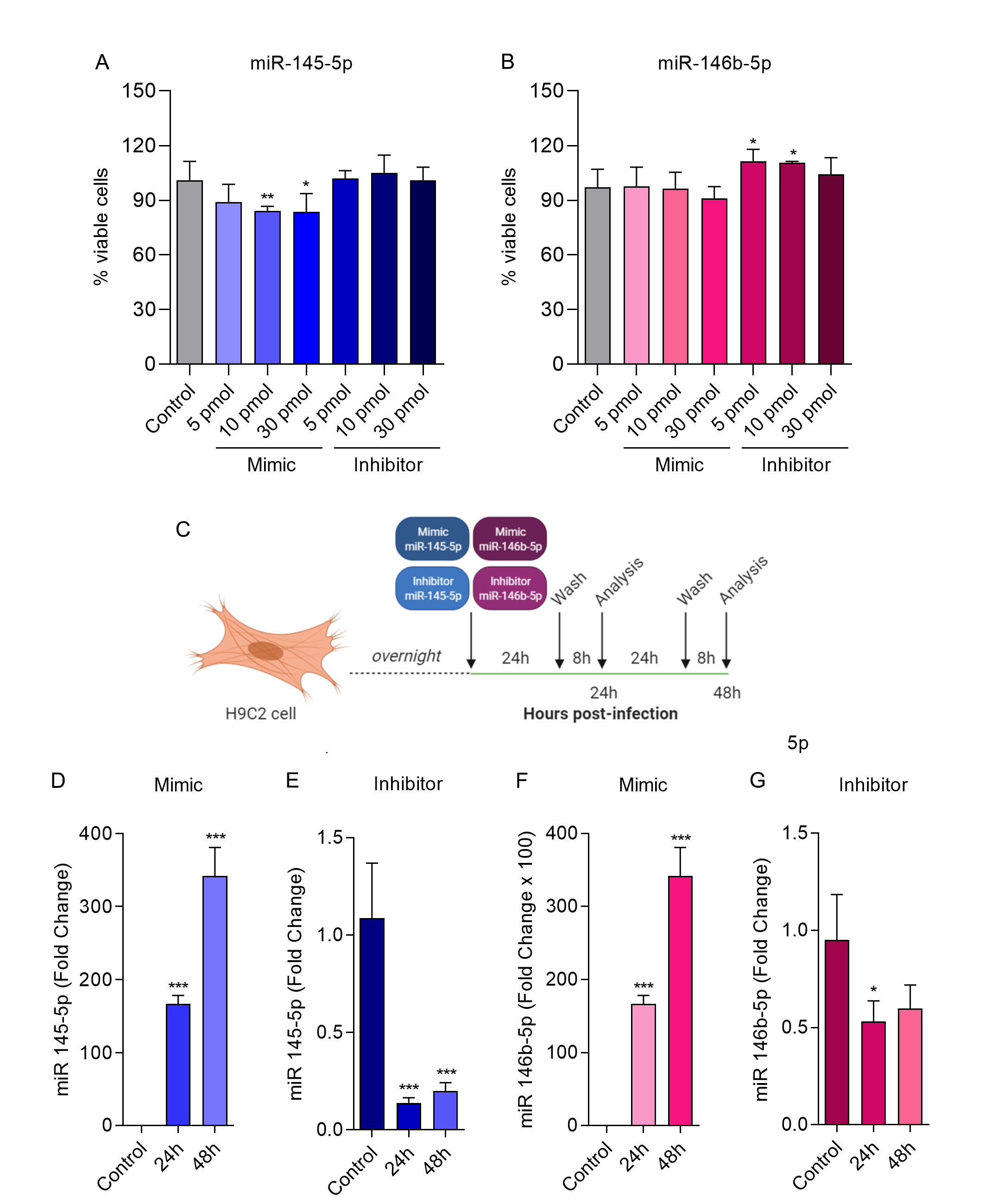

Supplement: Supplementary file 6 — Supplementary Figure 5. [file 41598_2022_5493_MOESM6_ESM.tif]
